# Tardive Blepharospasm May Respond to Bilateral Pallidal Deep Brain Stimulation

**DOI:** 10.5334/tohm.594

**Published:** 2021-03-18

**Authors:** Virgilio Gerald H. Evidente, Francisco A. Ponce, Maris H. Evidente, Margaret Lambert, Robin Garrett

**Affiliations:** 1 Movement Disorder Center of Arizona, Scottsdale, AZ, US; 2 Barrow Neurological Institute, Phoenix, AZ, US

**Keywords:** Blepharospasm, deep brain stimulation, DBS, pallidal DBS, GPi, Tardive

## Abstract

**Background:**

To date, there have been no reports of tardive blepharospasm being treated with deep brain stimulation (DBS), though there have been two reports of focal blepharospasm responding favorably to bilateral pallidal DBS.

**Case:**

A 34 year old man with tardive blepharospasm that was refractory to oral medications as well as botulinum toxin types A and B underwent bilateral pallidal DBS under general anesthesia. He had significant improvement of his severe blepharospasm by one and half months post-DBS which was sustained at last follow-up 30 months post-DBS. The best programming parameters included pulse widths of 90–100 µsec, frequencies of 140–150 Hz, and stimulating the ventral contacts in each side.

**Conclusion:**

Our case represents the first report of medically refractory tardive blepharospasm responding favorably to bilateral pallidal DBS.

## Introduction

Blepharospasm is the second most common focal dystonia that arises from excessive contraction of the orbicularis oculi and adjacent muscles including the procerus and corrugator [1]. It usually starts as frequent blinking, and progresses to muscle spasms that lead to forceful eye closure and functional blindness in severe cases. Blepharospasm that occurs with mid-facial, lower facial, or oromandibular dystonia is often referred to as Meige syndrome. Although milder cases may respond to anticonvulsants, benzodiazepines or muscle relaxants, moderate to severe cases often require botulinum toxin injections. Currently, two neurotoxins are FDA- approved in the US for blepharospasm including onabotulinumtoxinA and incobotulinumtoxinA, although there are studies as well of abobotulinumtoxinA showing equivalent efficacy to onabotulinumtoxinA [2,3], as well as rimabotulinumtoxinB being effective for benign essential blepharospasm resistant to botulinum toxin A [4]. However, some patients become resistant with chronic injections. Although there are several published studies that show the efficacy of pallidal deep brain stimulation (DBS) for Meige syndrome [5,6], there are only two case reports of pallidal DBS for focal blepharospasm and no reported cases of tardive blepharospasm being managed with DBS [7,8]. We describe a case of tardive blepharospasm that responded long-term to bilateral pallidal DBS.

## Case Report

A 35-year old right-handed Caucasian man presented to our center for evaluation of eyelid spasms. At age 6, he first developed tics consisting of facial grimacing, arm jerking, blinking, tongue protrusion and producing clicking sounds. The movements were associated with an urge and satisfaction, and were suppressible though causing inner stress when suppressed. His tics improved with age, and disappeared at age 15. In his late 20’s, he developed severe depression and anxiety and started seeing a psychiatrist. He was then put on various drugs including risperidone, olanzapine, and quetiapine. By age 33, he developed involuntary non-suppressible eyelid blinking and eyelid spasms that were worsened by bright lights, stress, anxiety and poor sleep. The eyelid spasms progressively worsened over several months to the point where he could only drive short distances. He also struggled tremendously at work as a radiology technician to keep his eyes open and focused. His psychiatrist at that time thought he may be having a recurrence of his tics and put him on pimozide with poor benefit. By the time he was first seen in our center at age 35, he was on a regimen of lurasidone and clonazepam both for his mood disorder and eyelid spasms, as well as mirtazapine and sertraline for his depression. He also had just received onabotulinumtoxinA shots to his eyelids with only brief relief of a few weeks.

**Figure 1:**
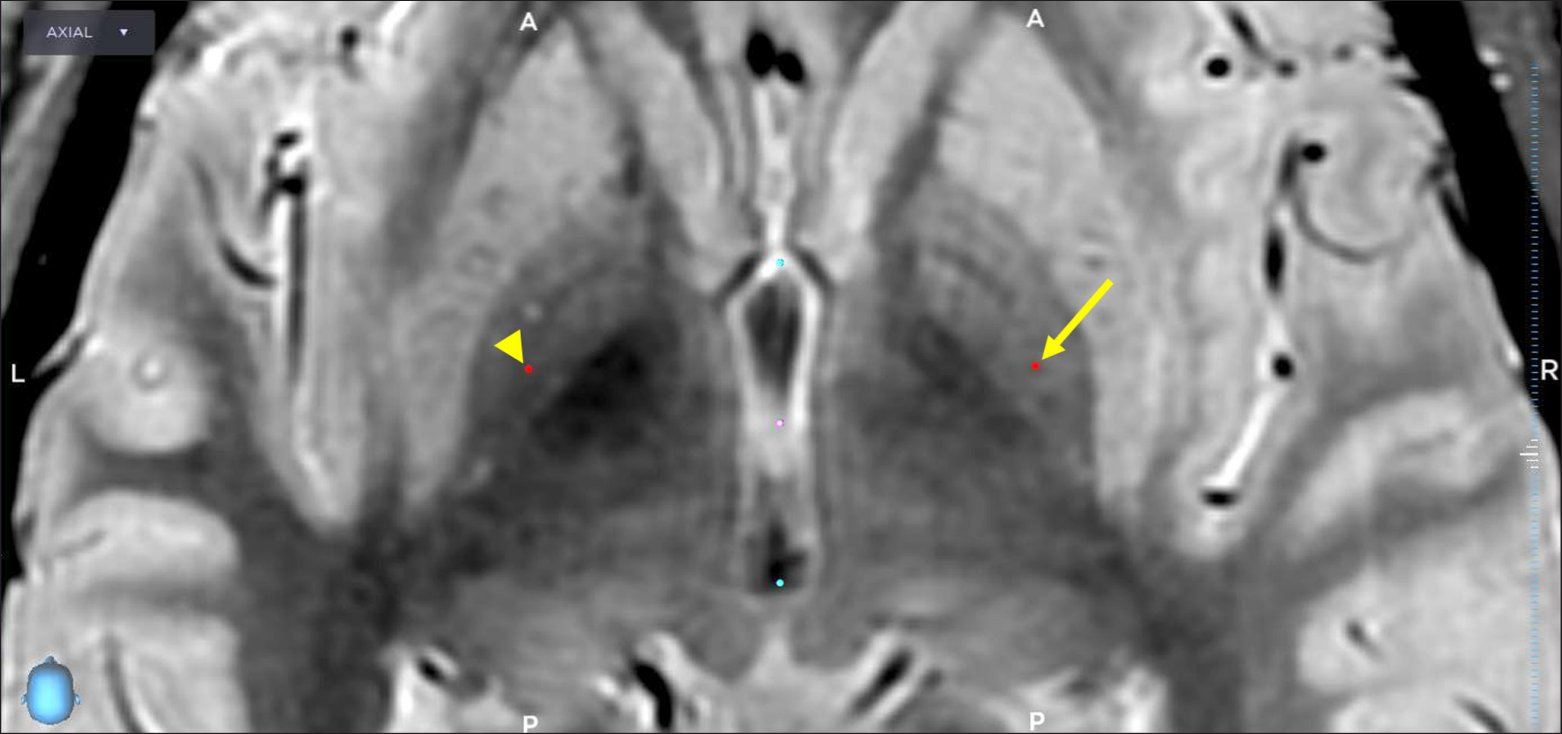
Proton density weighted images obtained on a 3-Tesla MRI demonstrates the anatomical target on the left (arrowhead) and right (arrow) corresponding to Talairach coordinates of (–21, 4.5, 0) and (21.25, 4.75, 0) respectively.

**Figure 2:**
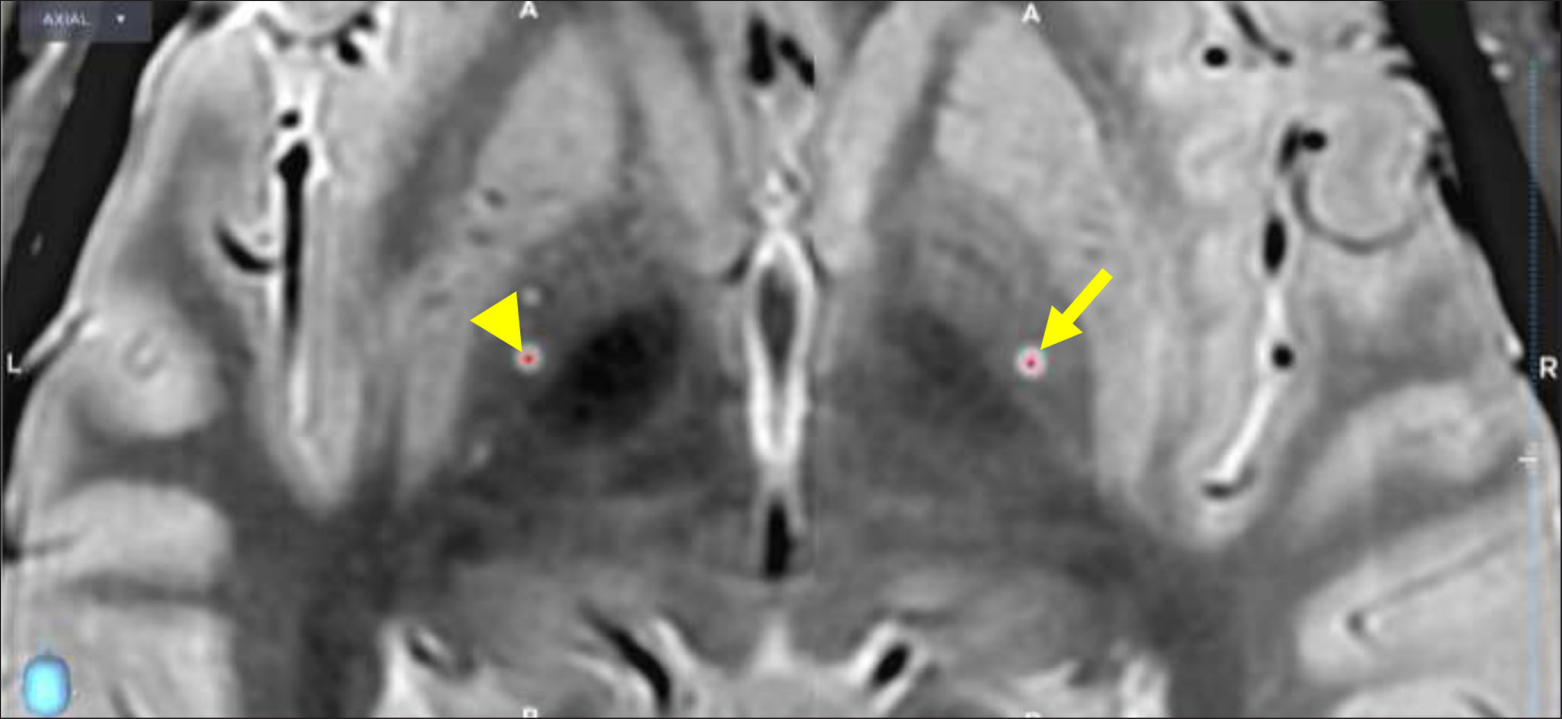
An intraoperative CT scan, taken immediately after bilateral lead placement, is merged with the preoperative MRI to assess stereotactic error and to visualize the contact location relative to the surrounding anatomy. Visible in this figure are contact 2 on the left (i.e., third contact from the bottom, arrowhead) and contact 9 on the right (i.e., second contact from the bottom, arrow). Contact 2 is centered at (–20.86, 4.04, –1.25), representing a radial error of 0.2 mm off of the surgical plan. Contact 9 is centered at coordinates (21, 4.25, –0.57), representing a radial error of 0.3 mm off of the surgical plan. Both contacts are positioned medial to the border between the globus pallidus interna and the globus pallidus externa.

Initial examination at age 35 revealed severe blepharospasm which was made worse by bright light (***Video 1***). His Burke-Fahn-Marsden (BFM) dystonia scale score was 4 and his Jankovic Rating Scale (JRS) for blepharospasm score was 6. He was thought to be suffering from tardive blepharospasm. He was then tried on up to 80 mg per day of valbenazine with poor benefit. Clonidine was added with poor results. Deutetrabenazine was introduced in lieu of valbenazine up to 48 mg/day with only limited improvement. He was next treated with incobotulinumtoxinA injections with no benefit. He was then shifted to rimabotulinumtoxinB as it was felt that he may be resistant to botulinum toxin type A. He initially responded to rimabotulinumtoxinB injections though the benefit waned after the second set of injections. At that point, he underwent bilateral pallidal DBS under general anesthesia. The rationale for general anesthesia was the patient’s anxiety about being awake for surgery. Preoperative imaging was obtained on a 3-T MRI, and a proton density sequence (2-mm slices) was used for GPi targeting. The Talairach coordinates were (–21, 4.5, 0) on the left and (21.25, 4.75, 0) on the right (***Figure 1***), with placement of the most distal contact at the ventral border of the GPi above the optic tract (projected 5.5 mm and 5.0 mm beyond target, respectively). The operation was performed using the Leksell G frame (Elekta Instrument AB, Stockholm, Sweden), and intraoperative imaging was obtained using the Samsung BodyTom CT scanner. Scans were obtained for frame registration and again immediately following the insertion of the second lead. The patient was implanted with bilateral Medronic quadripolar 3387 DBS electrodes. Post-lead CT scan showed a stereotactic error of 0.2 mm on the left and 0.3 mm on the right (***Figure 2***). Further details of the operative procedure have been previously published [9]. Two weeks later, a Medtronic Activa PC implantable pulse generator was placed in the left subclavicular area and connected to the leads via a cervical extension wire. Programming was initiated the week after surgery and done roughly every week initially, then every few weeks thereafter. He started to note mild improvement by the second week, and by the sixth week of programming, his eyelid spasms were practically gone (***Video 2***). His BFM and JRS scores were zero. When his stimulator was switched off at the sixth week, there was prompt return of blepharospasm within a few minutes (***Video 3***). The programming parameters at the sixth week post-DBS were the following; for the left GPi, settings were case (+), 1 (–), 3 volts, 90 µsec and 150 Hz; for the right GPi, settings were case (+), 9 (–), 3 volts, 100 µsec and 150 Hz. He continued to be programmed monthly with the amplitudes being increased with time as he would have breakthrough periods where his eyelid spasms would come out. By his seventh month post-DBS, his blepharospasm had stabilized and was well controlled. His BFM and JRS scores were 0. His programming settings at this point were the following: for the left GPi, settings were 3 (+), 1 (–), 3.5 volts, 90 µsec and 150 Hz; for the right GPi, settings were 11 (+), 8 and 9 (–), 3.6 volts, 100 µsec and 150 Hz. He was last evaluated at two and a half years post-DBS with still no blepharospasm noted (***Video 4***). His programming settings were the following: for the right GPi, settings were 3 (+), 1 (–), 3.6 volt, 90 µsec and 140 Hz; for the left GPi, settings were 11 (+), 8 and 9 (–), 3.6 volts, 100 µsec and 140 Hz.

**Video 1:** Patient with severe blepharospasm before DBS.

**Video 2:** One and a half months post-DBS with stimulator ON. Blepharospasm is markedly improved.

**Video 3:** One and a half months post-DBS with stimulator OFF. There was prompt return of eyelid spasms on switching the stimulator OFF.

**Video 4:** Two and a half years post-DBS with stimulator ON. There was sustained marked improvement of blepharospasm.

## Discussion

Santos et al reported in 2016 the first case of pallidal stimulation successfully done on focal blepharospasm, with their patient responding best to interleaving stimulation using pulse widths of 60–90 µsec and frequencies of 125–130 Hz [7]. This was soon followed in the same year by the report of Yamada et al of focal blepharospasm responding to bilateral pallidal DBS but at a very long pulse width of 450 µsec and frequency of 130 Hz [8]. Our current report is the third case of focal blepharospasm and first case of tardive blepharospasm responding to bilateral pallidal DBS, with complete resolution of the patient’s blepharospasm at pulse widths of 90–100 µsec and frequencies of 140–150 Hz. Studies on the effect of programming parameters on dystonia have noted that frequently used initial settings include short pulse widths (60–120 µsec), medium to high frequencies (130–185 Hz) and active or negative contacts in the ventral part of the GPi [10]. Some patients with dystonia, however, may benefit best from much wider pulse widths of up to 450 µsec, as in the case described by Yamada et al [8]. One study has shown that there were no significant differences in control of dystonia when comparing pulse widths of 60, 120, and 450 µsec [11]. The main advantages of narrow pulse widths include less current spread to neighboring structures, less side effects, and slower battery depletion. All three cases responded to the same medium to high frequencies commonly employed in Essential Tremor or Parkinson’s disease, and that frequencies greater than 185 Hz may not be necessary and may lead to faster depletion of the pulse generator.

Neurophysiological studies of the GPi have shown a ventral-to-dorsal somatotopic arrangement of neurons corresponding to the orofacial, upper limb and lower limb regions [12]. Our patient’s eyelid spasms responded best to stimulation of the ventral contacts in the GPi where the orofacial fibers are presumably located. The peak benefit of stimulation was noted by our patient at six weeks post-DBS, though consistent stable response was achieved by seven months post-DBS. This is similar to reports of increasing benefit of pallidal DBS on dystonia over several months, which has been attributed to neuroplasticity changes that take place in the basal ganglia with time [13]. The effect of pallidal DBS is enduring for controlling blepharospasm, with our case having complete resolution of symptoms at 30 months follow-up and the case described by Santos et al having sustained benefit at 21 months [7]. Thus, tolerance to the programming parameters we employed was not observed at two and a half years post-DBS.

Our case demonstrates that pallidal DBS is effective long-term in alleviating medically refractory focal tardive blepharospasm. Our case also suggests that stimulation of the ventral GPi is optimal in alleviating focal blepharospasm and that relatively narrow pulse widths and high frequencies control the eyelid spasms effectively. Larger cases series or controlled studies are required to confirm our observations.

## References

[1] Evidente VG, Adler CH. Hemifacial spasm and other craniofacial movement disorders. Mayo Clin Proc. 1998; 73: 67–71. DOI: 10.1016/S0025-6196(11)63621-5 9443681

[2] Kenney C, Jankovic J. Botulinum toxin in the treatment of blepharospasm and hemifacial spasm. J Neural Transm (Vienna). 2008; 115: 585–591. DOI: 10.1007/s00702-007-0768-7 17558461

[3] Evidente VG, Truong D, Jankovic J, Comella CL, Grafe S, Hanschmann A. IncobotulinumtoxinA (Xeomin®) injected for blepharospasm or cervical dystonia according to patient needs is well tolerated. J Neurol Sci. 2014; 346: 116–120. DOI: 10.1016/j.jns.2014.08.004 25186131

[4] Dutton JJ, White JJ, Richard MJ. Myobloc for the treatment of benign essential blepharospasm in patients refractory to botox. Ophthalmic Plast Reconstr Surg. 2006; 22: 173–177. DOI: 10.1097/01.iop.0000217382.33972.c4 16714924

[5] Wang X, Zhang Z, Mao Z, Yu X. Deep brain stimulation for Meige syndrome: a meta-analysis with individual patient data. J Neurol. 2019; 266: 2646–2656. DOI: 10.1007/s00415-019-09462-2 31302747

[6] Lyons MK, Birch BD, Hillman RA, Boucher OK, Evidente VG. Long-term follow-up of deep brain stimulation for Meige syndrome. Neurosurg Focus. 2010; 29: E5. DOI: 10.3171/2010.4.FOCUS1067 20672922

[7] Santos AF, Veiga A, Augusto L, Vaz R, Rosas MJ, Volkmann J. Successful treatment of blepharospasm by pallidal neurostimulation. Mov Disord Clin Pract. 2016; 3: 409–411. DOI: 10.1002/mdc3.12297 30713932 PMC6353506

[8] Yamada K, Shinojima N, Hamasaki T, Kuratsu J. Pallidal stimulation for medically intractable blepharospasm. BMJ Case Rep. 2016; 2016. DOI: 10.1136/bcr-2015-214241 PMC4823533 27033410

[9] Chen T, Mirzadeh Z, Chapple KM, Lambert M, Shill HA, Moguel-Cobos G, et al. Clinical outcomes following awake and asleep deep brain stimulation for Parkinson disease. J Neurosurg. 2018; 65: 1–12. DOI: 10.3171/2017.8.JNS17883 29547091

[10] Koeglsperger T, Palleis C, Hell F, Mehrkens JH, Bötzel K. Deep brain stimulation programming for movement disorders: current concepts and evidence-based strategies. Front Neurol. 2019; 10: 410. DOI: 10.3389/fneur.2019.00410 31231293 PMC6558426

[11] Vercueil L, Houeto JL, Krystkowiak P, Lagrange C, Cassim F, Benazzouz A, et al; Spidy GROUP (French Pallidal stimulation Group for dystonia). Effects of pulse width variations in pallidal stimulation for primary generalized dystonia. J Neurol. 2007; 254: 1533–1537. DOI: 10.1007/s00415-007-0578-8 17597333

[12] Nambu A. Somatotopic organization of the primate basal ganglia. Front Neuroanat. 2011; 5: 26. DOI: 10.3389/fnana.2011.00026 21541304 PMC3082737

[13] Ruge D, Tisch S, Hariz MI, Zrinzo L, Bhatia KP, Quinn NP, et al. Deep brain stimulation effects in dystonia: time course of electrophysiological changes in early treatment. Mov Disord. 2011; 26: 1913–1921. DOI: 10.1002/mds.23731 21547950 PMC3174341

